# Establishment and Characterization of *TGFBI* R124L iPSCs Derived from a Reis–Bücklers Corneal Dystrophy Family

**DOI:** 10.3390/cells15212025

**Published:** 2026-11-06

**Authors:** Momoko Kawasaki-Takahashi, Ryo Tomemori, Yoshikazu Matsuoka, Tatsuya Kanagawa, Yoshiki Yasukochi, Koichiro Higasa, Hisanori Imai, Kaoru Araki-Sasaki, Hirofumi Hitomi

**Affiliations:** 1 Department of Ophthalmology, Kansai Medical University, Hirakata 573-1010, Osaka, Japan; momoko2918@outlook.jp (M.K.-T.); tomemori.ryo@kmu.ac.jp (R.T.); imai.his@kmu.ac.jp (H.I.); 2 Department of iPS Stem Cell Regenerative Medicine, Kansai Medical University, Hirakata 573-1010, Osaka, Japan; m231009@med.kmu.ac.jp (T.K.); hitomi.hir@kmu.ac.jp (H.H.); 3 Department of Genome Analysis, Institute of Biomedical Science, Kansai Medical University, Hirakata 573-1010, Osaka, Japan; yasukouchi.yos@kmu.ac.jp (Y.Y.); higasa.koi@kmu.ac.jp (K.H.)

**Keywords:** Reis–Bücklers corneal dystrophy, *TGFBI*, R124L, induced pluripotent stem cells, ocular surface organoid, corneal dystrophy, disease modeling

## Abstract

**Highlights:**

**What are the main findings?**

• New iPSC lines were established from an affected daughter and father and an unaffected mother in the same RBCD family.
• The two affected lines retained the *TGFBI* R124L variant, and all three lines expressed pluripotency markers, demonstrated trilineage differentiation potential, and developed early SEAM-like morphology under induction conditions.

**What are the implications of the main findings?**

• This family set enables comparison of the daughter-derived *TGFBI* R124L line with a genetically related unaffected maternal control and includes an independently derived affected paternal line.
• The lines can be used to study TGFBIp deposition and epithelial–stromal interactions after corneal epithelial and stromal differentiation.

**Abstract:**

Reis–Bücklers corneal dystrophy (RBCD) is an early-onset *TGFBI*-related corneal dystrophy associated with the *TGFBI* R124L variant. It is characterized by recurrent corneal epithelial erosion, hyaline-like deposits involving Bowman’s layer and the anterior stroma, and progressive subepithelial corneal opacity. However, how mutant *TGFBI* protein (TGFBIp) accumulates in the cornea remains unclear. In this study, we established induced pluripotent stem cells (iPSCs) from two affected members of an RBCD family and one unaffected family member. The patient-derived lines retained the R124L variant, whereas the line established from the unaffected mother did not. All three lines showed pluripotency and trilineage differentiation, had normal karyotypes, and were confirmed by STR analysis to match their donors. After 4 weeks of SEAM induction, all three iPSC lines developed early SEAM-like cultures containing PAX6- and p63-positive cells, demonstrating their capacity for early differentiation toward the corneal epithelial lineage. TGFBIp was also detected in extracellular and pericellular regions by immunofluorescence. In silico analysis detected no major structural difference between WT and R124L TGFBIp in the high-confidence CROPT and FAS1 domain regions, whereas surface analysis showed local changes in electrostatic potential and molecular lipophilicity around residue 124. These lines can be used to study TGFBIp deposition and epithelial–stromal interactions in RBCD.

## 1. Introduction

*TGFBI*-related corneal dystrophies are a group of autosomal dominant corneal disorders caused by mutations in the transforming growth factor beta-induced (*TGFBI*) gene and exhibit diverse clinical phenotypes depending on the mutation site. *TGFBI* encodes TGFBIp, an extracellular matrix protein that is abundant in the human cornea, particularly in the corneal stroma [1,2]. R124 and R555 are recognized as major mutation hotspots, and variants such as R124H, R124C, R124L, R555W, and R555Q are among the major targets of molecular diagnosis [2,3,4,5]. Reis–Bücklers corneal dystrophy (RBCD), originally described by Reis and Bücklers [6,7], is typically associated with the *TGFBI* R124L mutation [8,9,10,11]. RBCD is characterized by recurrent corneal epithelial erosion, progressive subepithelial opacity, and deposits involving Bowman’s layer and extending into the anterior stroma [8,9,10]. In edition 3 of the International Committee for Classification of Corneal Dystrophies (IC3D) classification, RBCD is classified as a *TGFBI*-related corneal dystrophy based on integrated clinical, pathological, and genetic findings [8]. RBCD is considered rare, although its population prevalence has not been established. The R124L variant was not detected among 129,933 participants in a South Korean genetic screening study. In a Chinese cohort, R124L was identified in only two of 56 genetically confirmed corneal dystrophy patients (3.57%) [12,13].

Although hyaline-like deposits are known to accumulate from the subepithelial region to Bowman’s layer and the anterior stroma in RBCD, the mechanisms of deposit formation remain poorly understood [8,9,10,14,15]. In particular, it remains unclear whether the deposits are mainly produced by corneal epithelial cells, stromal cells, or through interactions between these cell types. It is also unknown how the *TGFBI* R124L mutation affects TGFBIp kinetics and leads to protein aggregation. Different *TGFBI* mutations may affect TGFBIp kinetics in different ways [14,15] and are associated with distinct disease phenotypes. Despite these differences, TGFBIp accumulation is considered to contribute to disease pathogenesis across *TGFBI*-related corneal dystrophies. However, the molecular mechanisms of deposit formation, the relationship between mutant TGFBIp and the extracellular matrix, and potentially targetable molecular processes require further investigation [14,15,16]. Therefore, reproducible disease models using patient-derived cells are needed to analyze the long-term pathogenesis of RBCD.

Patient-derived induced pluripotent stem cells (iPSCs) are valuable for modeling rare inherited disorders and for testing candidate interventions in disease-relevant cell types [17]. In ophthalmology, self-formed ectodermal autonomous multizone (SEAM) induction from human iPSCs enables two-dimensional reconstruction of ocular surface lineages, including corneal epithelium [18,19]. For RBCD, family-derived iPSCs that retain the R124L mutation would offer a source of cells for later analyses of corneal epithelial and stromal differentiation and TGFBIp deposition.

Here, we report the generation and initial characterization of peripheral blood-derived iPSCs from an RBCD family. We examined mutation retention, pluripotency marker expression, trilineage differentiation, karyotype, and donor identity, as well as morphology and marker expression following SEAM induction. Because residue 124 may influence intermolecular or extracellular matrix interactions, we additionally compared the predicted overall structures of WT and R124L TGFBIp and examined whether the R124L substitution alters local surface properties, thereby generating hypotheses for subsequent studies using corneal cells differentiated from the established iPSCs.

## 2. Materials and Methods

### 2.1. Ethics Approval and Informed Consent

This study was approved by the Institutional Review Board of Kansai Medical University (approval No. 2023124). All procedures were performed in accordance with the tenets of the Declaration of Helsinki. Written informed consent was obtained from the adult participants and from a parent of the minor participant for peripheral blood collection, iPSC generation, genetic analysis, and the use of the resulting samples and data for research and publication. Written informed consent was obtained from the minor participant.

### 2.2. Participants and Peripheral Blood Collection

This study included three members of a family in which Reis–Bücklers corneal dystrophy (RBCD) was associated with the *TGFBI* R124L variant. The proband was a 12-year-old girl, and her father and mother were both 45 years old. The proband and her father were affected by RBCD, whereas the mother was an unaffected family control with no clinically apparent corneal lesions. Peripheral blood (12 mL) was collected from each participant and used for *TGFBI* genetic analysis and iPSC generation. The clinical information of the participants and the established iPSC lines is summarized in Table 1.

### 2.3. Genomic DNA Extraction and Sanger Sequencing of TGFBI

Genomic DNA was extracted from peripheral blood mononuclear cells (PBMCs) using the DNeasy Blood & Tissue Kit (QIAGEN, Hilden, Germany), and the region containing the *TGFBI* variant NM_000358.3:c.371G>T, predicted to result in NP_000349.1:p.(Arg124Leu), was amplified by polymerase chain reaction (PCR). After purification of the PCR products, the nucleotide sequence was analyzed by Sanger sequencing to determine the presence or absence of the variant. Variant nomenclature was based on the *TGFBI* reference transcript NM_000358.3 and the corresponding protein sequence NP_000349.1. Amino acid numbering refers to the full-length TGFBIp precursor, including the N-terminal signal peptide. The primer sequences used in this study are listed in Table 2.

### 2.4. Generation of iPSCs from Peripheral Blood Mononuclear Cells

iPSCs were generated from PBMCs using a nonviral episomal vector method described by Okita et al. [20], with minor modifications. Briefly, PBMCs were cultured for 7 days on mesenchymal stem cell (MSC) feeders established from commercially available human bone marrow mononuclear cells (STEMCELL Technologies, Vancouver, BC, Canada) in StemPro-34 SFM medium (Thermo Fisher Scientific, Waltham, MA, USA) supplemented with 10% fetal bovine serum (FBS; Cytiva, Marlborough, MA, USA), thrombopoietin (TPO; Proteintech, Rosemont, IL, USA), stem cell factor (SCF; Thermo Fisher Scientific), granulocyte colony-stimulating factor (G-CSF; Thermo Fisher Scientific), Flt3 ligand (FL; Proteintech), interleukin-3 (IL-3; Thermo Fisher Scientific), interleukin-6 (IL-6; Thermo Fisher Scientific), and GlutaMAX (Thermo Fisher Scientific) to expand hematopoietic stem/progenitor cells. The cells were then collected and electroporated with the plasmids pCE-hOCT3/4 (Addgene, Watertown, MA, USA; #41813), pCE-hSK (Addgene; #41814), pCE-hUL (Addgene; #41855), pCE-mp53DD (Addgene; #41856), and pCXB-EBNA1 (Addgene; #41857). The cells were suspended in the buffer supplied with the Human T Cell Nucleofector Kit (Lonza, Cologne, Germany), and electroporation was performed using a Nucleofector 2b device (Lonza, Cologne, Germany) with program Q-003. After gene transfer, the expanded hematopoietic stem/progenitor cells were plated on 6-well plates coated with iMatrix-511 (Matrixome, Inc., Osaka, Japan; laminin-511 E8 fragment) [21] and cultured again in 1.5 mL of hematopoietic medium. On days 2, 4, and 6, 1.5 mL of StemFit AK02 medium (Ajinomoto, Tokyo, Japan) was added, and on day 8, the medium was completely replaced with StemFit AK02 medium. Emerging iPSC-like colonies were visually selected under a microscope and manually picked.

The established iPSCs were expanded in StemFit AK02 medium. All cultures were maintained at 37 °C in 5% CO_2_. Cells were passaged with Accutase (Innovative Cell Technologies, Inc., San Diego, CA, USA) when they reached 70–90% confluence. Stocks of each iPSC line were prepared at passage 3, and all characterization data for the established iPSCs were obtained using passage 4 cells. Mycoplasma contamination was assessed at passage 4 using the MycoAlert Mycoplasma Detection Kit (Lonza, Basel, Switzerland) according to the manufacturer’s instructions.

### 2.5. Confirmation of the TGFBI c.371G>T Variant in Established iPSCs

Genomic DNA was extracted from each established donor-derived iPSC line, and the region containing the *TGFBI* c.371G>T variant was amplified by PCR as described for peripheral blood-derived DNA. Sanger sequencing was used to confirm retention of the variant in patient-derived iPSCs and its absence in iPSCs derived from the unaffected control. Six independent iPSC colonies were analyzed for each donor. One colony-derived line from each donor was selected for subsequent characterization and differentiation experiments.

### 2.6. Flow Cytometry Analysis of Pluripotency Markers

Pluripotency-associated surface marker expression was analyzed by flow cytometry. iPSCs were dissociated into single cells using Accutase and, after washing, stained with antibodies against SSEA-4 (BioLegend, San Diego, CA, USA) and TRA-1-60 (BioLegend). Dead cells were excluded using 7-aminoactinomycin D (7-AAD, BioLegend). Cells were then analyzed on an Attune NxT flow cytometer (Thermo Fisher Scientific, Waltham, MA, USA). Information on the antibodies used in this study is provided in Table 3.

For analysis, the cell population was selected based on forward and side scatter, followed by doublet exclusion and dead-cell exclusion. The percentage of cells positive for each pluripotency marker was then calculated. Unstained controls were used for gate setting. The gating strategies used for flow cytometric analysis are shown in Supplementary Figure S1.

### 2.7. Immunocytochemistry for Pluripotency Markers

iPSCs were cultured for 7–8 days on iMatrix-511-coated 4-well culture plates (Corning Incorporated, Corning, NY, USA) and then fixed with 10% neutral buffered formalin for 5 min at room temperature. After fixation, cells were washed with phosphate-buffered saline [PBS(-); Nacalai Tesque, Inc., Kyoto, Japan] and permeabilized with 0.1% Triton X-100/PBS(-). Blocking was performed using Blocking One (Nacalai Tesque, Inc., Kyoto, Japan), and cells were stained with fluorescently labeled antibodies against SSEA-4, OCT3/4, NANOG, and SOX2 (BioLegend, San Diego, CA, USA). Nuclei were counterstained with Hoechst 33342 (Dojindo Laboratories, Kumamoto, Japan). Antibody information is provided in Table 3.

Fluorescence images were acquired using a BZ-X810 fluorescence microscope (KEYENCE, Osaka, Japan). Imaging conditions were kept identical across samples.

### 2.8. Karyotype Analysis

Karyotype analysis was performed using passage 4 iPSCs. Chromosomal G-banding analysis was outsourced to SRL, Inc. (Tokyo, Japan), and karyotypes were described according to the International System for Human Cytogenomic Nomenclature (ISCN).

### 2.9. Short Tandem Repeat Analysis

Donor identity was assessed by short tandem repeat (STR) analysis. Genomic DNA was extracted from peripheral blood-derived cells and the corresponding iPSCs, and nine STR loci and the amelogenin sex-typing marker were analyzed. The analysis was outsourced to BEX Co., Ltd. (Tokyo, Japan). STR profiles and amelogenin results of peripheral blood mononuclear cells and iPSCs were compared.

### 2.10. Trilineage Differentiation and Quantitative PCR

Trilineage differentiation into ectodermal, mesodermal, and endodermal lineages was performed using the STEMdiff Trilineage Differentiation Kit (STEMCELL Technologies, Vancouver, BC, Canada) according to the manufacturer’s protocol.

After differentiation, total RNA was extracted from the cells using ISOGEN II (Nippon Gene, Tokyo, Japan), and cDNA was synthesized using the iScript cDNA Synthesis Kit (Bio-Rad Laboratories, Hercules, CA, USA). Quantitative PCR (qPCR) was performed using Power SYBR Green PCR Master Mix (Thermo Fisher Scientific) on a Rotor-Gene Q real-time PCR system (QIAGEN, Hilden, Germany) to analyze the expression of the ectodermal markers *PAX6* and *SOX1*, the endodermal markers *SOX17* and *FOXA2*, and the mesodermal markers *TBXT* and *TBX6*. *ACTB* was used as the sole reference gene. Relative expression was analyzed using the comparative Ct method [22]. Three independent differentiation experiments were performed for each donor-derived iPSC line; the undifferentiated and three differentiated conditions were matched within each experiment. Two technical qPCR replicates per assay were averaged at the Ct level before calculation. No Ct-based exclusion criterion was applied, and all measured Ct values, including those from low-expression conditions, were retained in the calculations. ΔCt was calculated as mean Ct (target gene) − mean Ct (*ACTB*) within each donor-derived line, experiment, and cell state. ΔΔCt was calculated relative to the undifferentiated condition of the same line within the same experiment, and relative expression is presented as 2^−ΔΔCt^. Amplification efficiencies were not evaluated using dilution-series standard curves; the assumption of comparable target and reference assay efficiencies was therefore not independently verified. *ACTB* Ct values are provided in Supplementary Table S1. Experiment-level Ct values for ACTB and the six target genes are provided in Supplementary Table S2. The primer sequences used are listed in Table 2.

### 2.11. SEAM Induction

Donor-derived iPSCs were dissociated into single cells and seeded onto iMatrix-511-coated 4-well plates at a density of 300 cells/well. SEAM induction was performed based on the methods described by Hayashi et al. [18,19], and the culture period was 4 weeks. The cultures were observed by phase-contrast microscopy to evaluate early SEAM-like morphology.

After 4 weeks of SEAM induction, cultures were washed with PBS and fixed with 4% paraformaldehyde in PBS for 15 min. Following three PBS washes, the cultures were permeabilized with 0.1% Triton X-100 in PBS for 15 min, washed three times, and blocked with 10% donkey serum (Merck Millipore, Temecula, CA, USA) in PBST (0.05% Tween 20 in PBS) for 30 min. The cultures were incubated overnight at 4 °C with primary antibodies against p63, PAX6, and TGFBIp diluted in blocking buffer. After three washes for 5 min each, the corresponding Alexa Fluor-conjugated donkey secondary antibodies were applied at a dilution of 1:200 for 1 h at room temperature in the dark. The cultures were then washed three times for 5 min each with PBST and counterstained with Hoechst 33342 (final concentration, 1 µg/mL) for 10 min at room temperature in the dark. Fluorescence images were acquired using a BZ-X810 fluorescence microscope. Antibody details are listed in Table 3.

### 2.12. In Silico Structural and Surface Analyses of the TGFBI R124L Variant

In silico structural and surface analyses were performed to compare WT and R124L TGFBIp and to examine local surface properties around residue 124. Full-length WT and R124L models were generated using AlphaFold 3 [23], and the top-ranked model from each prediction was used for structural comparison. Prediction confidence was evaluated using the per-atom predicted local distance difference test (pLDDT) values provided in the AlphaFold 3 output. Because the N-terminal region (residues 1–44) and C-terminal region (residues 633–683) showed lower prediction confidence, structural comparison was restricted to the CROPT domain and the four FAS1 domains, which showed high prediction confidence. The CROPT domain (residues 45–99) and the four FAS1 domains, FAS1-1 (residues 103–236), FAS1-2 (residues 240–371), FAS1-3 (residues 375–498), and FAS1-4 (residues 502–632), were superimposed using UCSF ChimeraX version 1.12 [24], and the Cα RMSD was calculated using 576 corresponding Cα atoms. Each domain was also analyzed separately.

Local surface properties around residue 124 were examined using chain A of the experimentally determined human TGFBIp structure (residues Gln43–Asn637) registered in the Protein Data Bank [25] under PDB ID: 5NV6 [26]. The WT model was prepared from chain A, and the R124L model was generated by replacing Arg124 with Leu using the ChimeraX swapaa command. Hydrogen atoms were added, and both models were energy-minimized using the ChimeraX minimize command with default settings. Solvent-excluded molecular surfaces were generated for the WT and R124L models. Coulombic electrostatic potentials were calculated after reassignment of Amber 20 recommended charges and atom types and mapped onto the protein surfaces using a red–white–blue scale ranging from −10 to +10 kcal/(mol·e). Molecular lipophilicity potential (MLP) was mapped using the ChimeraX mlp command with the lipophilicity palette ranging from −20 to +20. Local views were centered on residues 118–130. The WT and R124L models were displayed in the same orientation and at the same magnification.

### 2.13. Statistical Analysis

Trilineage qPCR data from three independent differentiation experiments per donor-derived iPSC line were analyzed descriptively. Individual points represent experiment-level relative expression values (2^−ΔΔCt^). Bars indicate the geometric mean of the fold-change values, with error bars corresponding to the mean (−ΔΔCt) ± SD (−ΔΔCt) after back-transformation. No formal statistical testing was performed.

## 3. Results

### 3.1. Clinical Features and Pedigree of a TGFBI R124L RBCD Family

**Figure 1:**
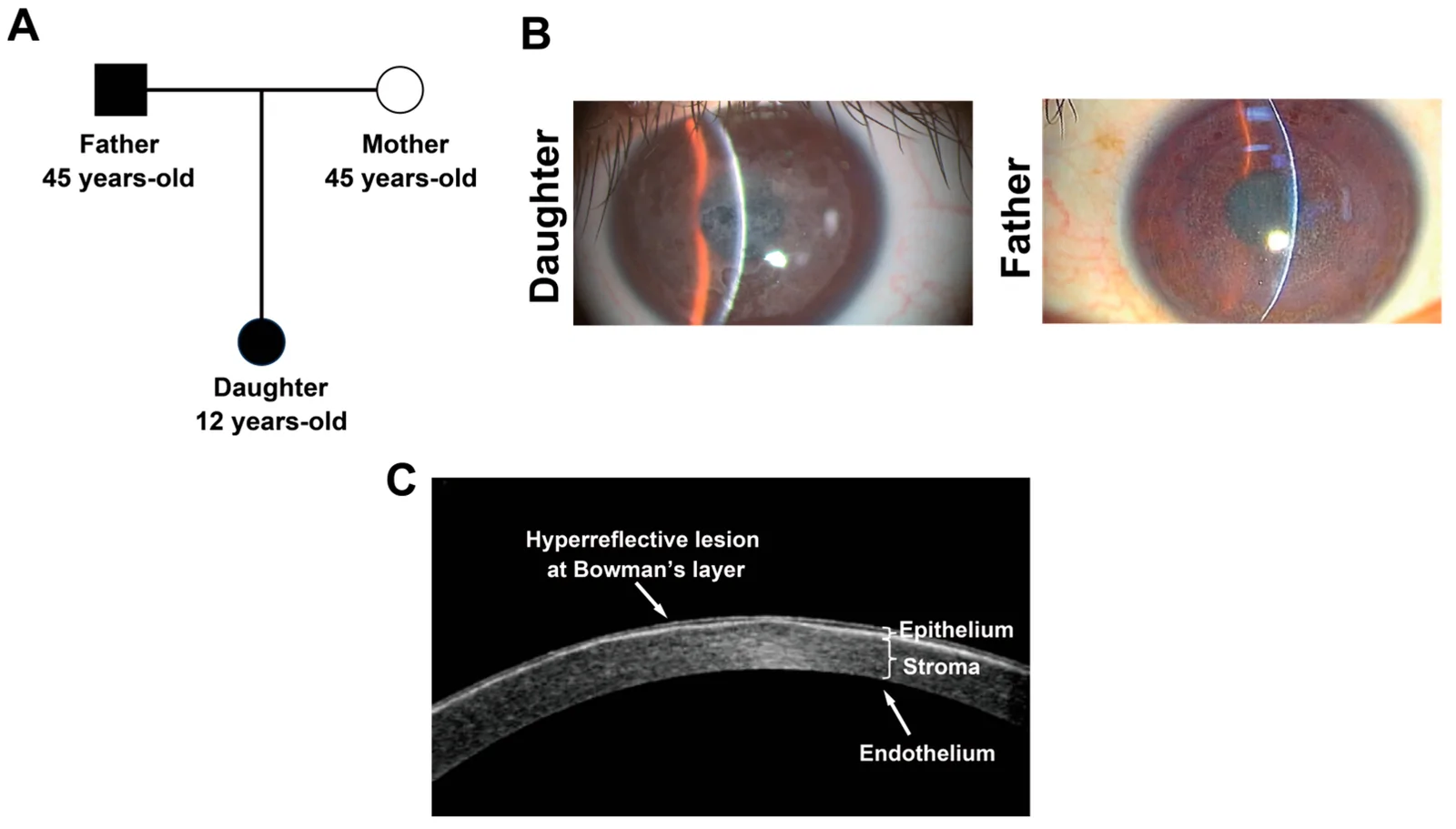
Clinical features of a family with Reis–Bücklers corneal dystrophy carrying the TGFBI R124L mutation: (**A**) Pedigree of the family analyzed in this study. The father and daughter were diagnosed with Reis–Bücklers corneal dystrophy (RBCD) and carried the TGFBI R124L mutation. The mother was an unaffected family control with no clinically apparent corneal lesions. The ages at blood collection were 45 years for the father, 45 years for the mother, and 12 years for the daughter. (**B**) Slit-lamp photographs of the left eyes of the affected daughter and father. Both patients showed corneal opacities extending from the subepithelial region to the anterior stroma, consistent with RBCD. (**C**) Anterior segment optical coherence tomography (OCT) image of the left eye of the daughter. Hyperreflective lesions were observed extending from the subepithelial region to Bowman’s layer and the anterior stroma.

The family analyzed in this work included a 12-year-old female proband with RBCD, her affected 45-year-old father, and her unaffected 45-year-old mother, who had no clinically apparent corneal lesions (Figure 1A). All three participants were enrolled for genetic analysis and iPSC generation.

The proband had refractory and frequent bilateral recurrent corneal epithelial erosion from the age of 3 years and was clinically diagnosed with RBCD. During early childhood, disposable soft contact lenses (DSCLs) were used to protect the corneal epithelium, and hard contact lenses (HCLs) were also used as appropriate in combination with DSCLs to treat amblyopia caused by astigmatism resulting from epithelial irregularities, thereby managing visual acuity. During the clinical course, deposits could be removed by curettage of the erosion base at the onset of corneal epithelial erosion, resulting in improved corneal transparency and a reduced frequency of epithelial erosion. After the school-age years, the frequency of epithelial erosion remained relatively stable, but subepithelial corneal opacities progressed; at age 12, corneal electrolysis was performed to reduce the opacities by dissolving the deposits with NaOH [27,28]. The father had similar corneal findings to the proband and had also been diagnosed with RBCD.

Slit-lamp examination showed corneal opacity consistent with RBCD in both the proband and her father (Figure 1B). Anterior segment optical coherence tomography (OCT) showed hyperreflective lesions extending from the subepithelial region to Bowman’s layer and the anterior stroma (Figure 1C). Imaging findings with similar layer localization have also been reported in previous studies using confocal microscopy and anterior segment OCT for Bowman’s layer dystrophies [29,30].

### 3.2. Generation of iPSCs from Peripheral Blood Cells of the RBCD Family

**Figure 2:**
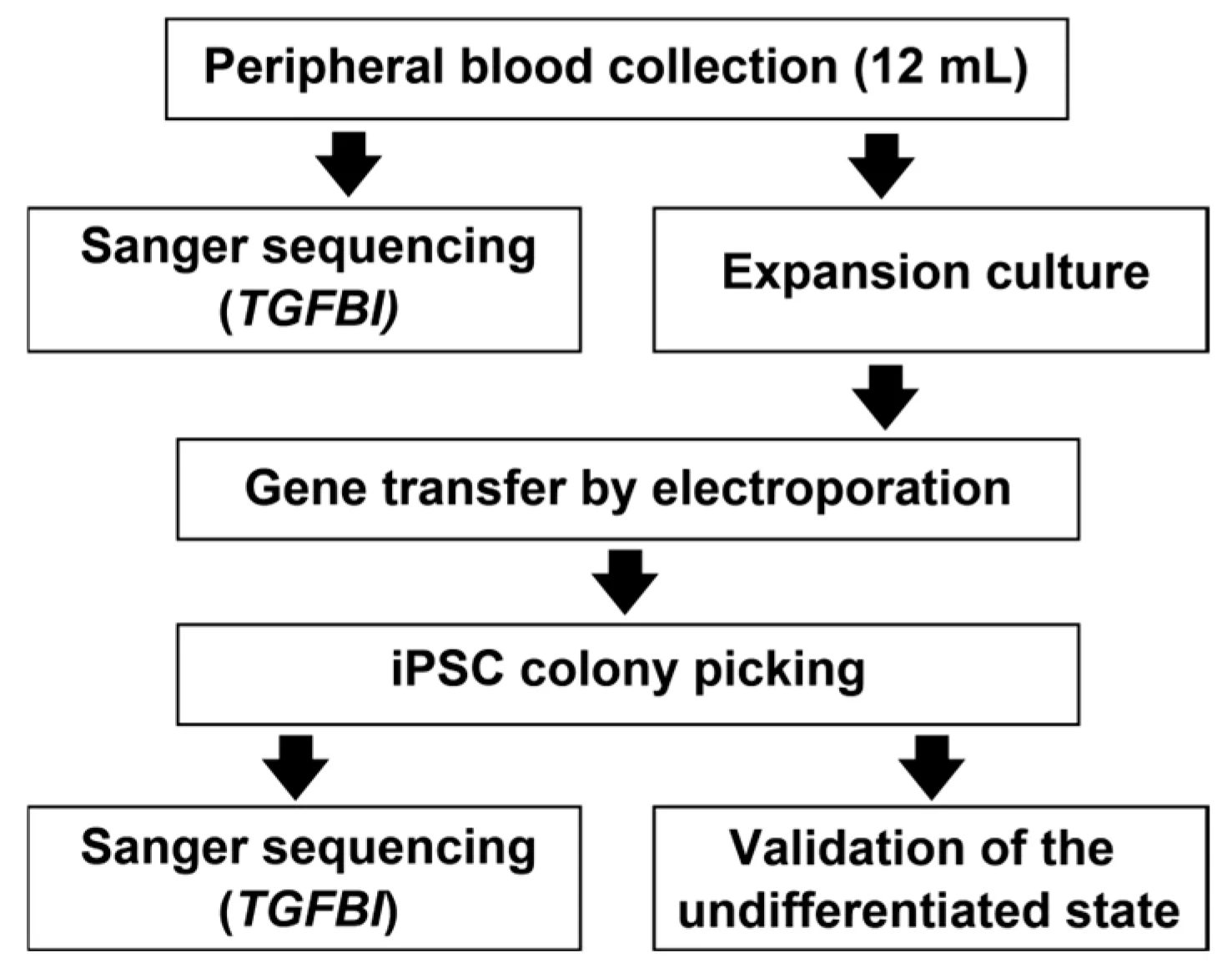
Workflow of iPSC generation and characterization. Peripheral blood (12 mL) was collected from each member of the RBCD family and used for *TGFBI* genetic analysis and iPSC generation. After expansion culture of peripheral blood-derived cells, episomal vectors were introduced by electroporation, and emerging iPSC-like colonies were picked. The established iPSCs were characterized for retention of the *TGFBI* R124L mutation, undifferentiated state, and subsequent cellular properties.

**Figure 3:**
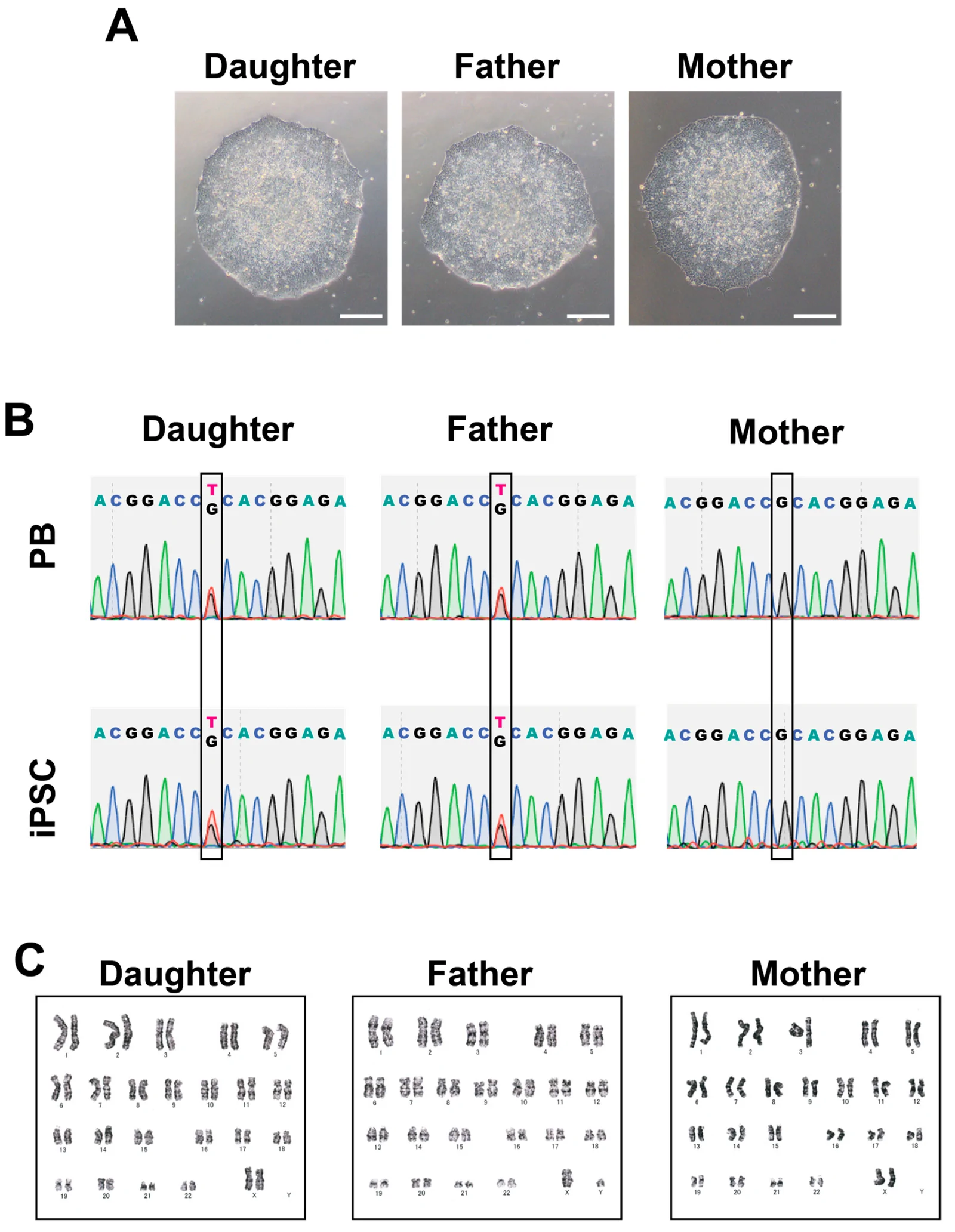
Establishment of iPSCs from the RBCD family, retention of the *TGFBI* mutation, and karyotype analysis: (**A**) Representative images of iPSC colonies derived from the daughter, father, and mother. Human iPSC-like colonies with clear borders were obtained from all donor-derived cells. Scale bar: 500 μm. (**B**) Sanger sequencing of *TGFBI* in peripheral blood-derived cells (PB) and corresponding iPSCs. In the affected daughter and father, the heterozygous *TGFBI* variant NM_000358.3:c.371G>T, predicted to result in NP_000349.1:p.(Arg124Leu), was confirmed in both PB-derived cells and iPSCs. The variant was not detected in the unaffected mother. Nucleotide peaks are color-coded as A (green), C (blue), G (black), and T (red). Black rectangles indicate the c.371 variant site. (**C**) G-banding karyotype analysis of iPSCs derived from the daughter, father, and mother. No apparent karyotypic abnormalities were detected in any iPSC line.

Peripheral blood (12 mL) was collected from each participant, and peripheral blood mononuclear cells were isolated. After short-term expansion culture, episomal vectors were introduced by electroporation. The workflow for iPSC generation is shown in Figure 2. iPSC-like colonies with flat morphology and well-defined borders were obtained from the daughter, father, and mother (Figure 3A).

### 3.3. Confirmation and Retention of the TGFBI c.371G>T Variant

Sanger sequencing of peripheral blood-derived genomic DNA detected the heterozygous *TGFBI* variant NM_000358.3:c.371G>T, predicted to result in NP_000349.1:p.(Arg124Leu), in the affected daughter and father, but not in the unaffected mother (Figure 3B, Table 1). The same variant was retained in daughter- and father-derived iPSCs but was not detected in mother-derived iPSCs. Similar results were obtained from six independent colonies analyzed for each donor-derived iPSC line.

### 3.4. Genomic Integrity and Donor IDENTITY of Established iPSCs

G-banding analysis showed a normal karyotype in all three iPSC lines (46, XX for the daughter and mother; 46, XY for the father; 20 metaphases analyzed per line) (Figure 3C).

STR profiles at all nine loci and amelogenin results were concordant between peripheral blood-derived cells and the corresponding iPSCs for each donor (Table 4), verifying the identity of the established iPSC lines.

### 3.5. Pluripotency Marker Expression in Established iPSCs

**Figure 4:**
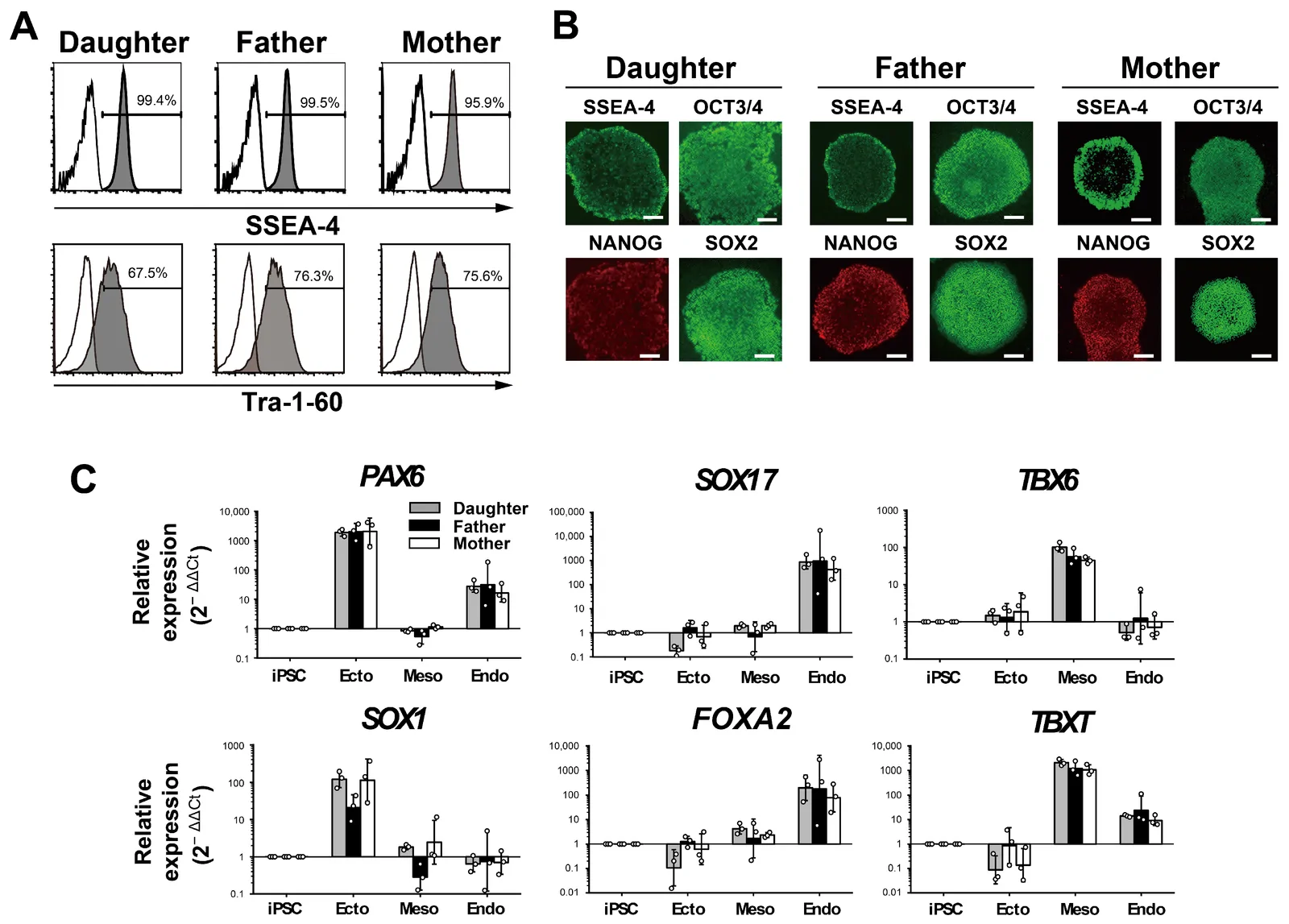
Evaluation of pluripotency marker expression and trilineage differentiation potential of established iPSCs: (**A**) Flow cytometric analysis of the pluripotency surface markers SSEA-4 and TRA-1-60 in iPSCs derived from the daughter, father, and mother. SSEA-4- and TRA-1-60-positive cells were detected in each iPSC line. The unfilled histograms indicate unstained controls, and the gray-filled histograms indicate antibody-stained cells. (**B**) Immunocytochemical analysis of pluripotency markers in iPSCs derived from the daughter, father, and mother. Each iPSC line expressed SSEA-4, OCT3/4, NANOG, and SOX2. In (**B**), fluorescence signals for SSEA-4, OCT3/4, and SOX2 are shown in green, whereas NANOG is shown in red. Scale bar: 100 μm. (**C**) qPCR analysis after trilineage differentiation. Relative expression is shown as 2^−ΔΔCt^, using undifferentiated iPSCs from the same experiment and donor-derived line as the calibrator. Individual points represent the three independent differentiation experiments per line. Bars represent the geometric mean of the fold-change values, and error bars indicate the interval obtained by back-transforming the mean (−ΔΔCt) ± SD (−ΔΔCt). No formal statistical testing was performed.

Flow cytometry detected SSEA-4- and TRA-1-60-positive cells in iPSCs derived from the daughter, father, and mother (Figure 4A). The SSEA-4-positive fractions were 99.4%, 99.5%, and 95.9%, whereas the TRA-1-60-positive fractions were 67.5%, 76.3%, and 75.6% in the daughter-, father-, and mother-derived lines, respectively. Immunocytochemistry also showed expression of SSEA-4, OCT3/4, NANOG, and SOX2 in all three donor-derived iPSC lines (Figure 4B).

### 3.6. Trilineage Differentiation Potential of Established iPSCs

Trilineage differentiation was performed using the STEMdiff Trilineage Differentiation Kit, and lineage marker expression was assessed by qPCR. Lineage-specific marker expression increased after induction toward the corresponding germ layer in all three iPSC lines (Figure 4C). In each of the three independent experiments for each donor-derived line, unnormalized Ct values for all six target genes were lower in the corresponding differentiated lineage than in the matched undifferentiated condition (Supplementary Table S2).

### 3.7. Early SEAM-like Morphology and Marker Expression Under Induction Conditions

**Figure 5:**
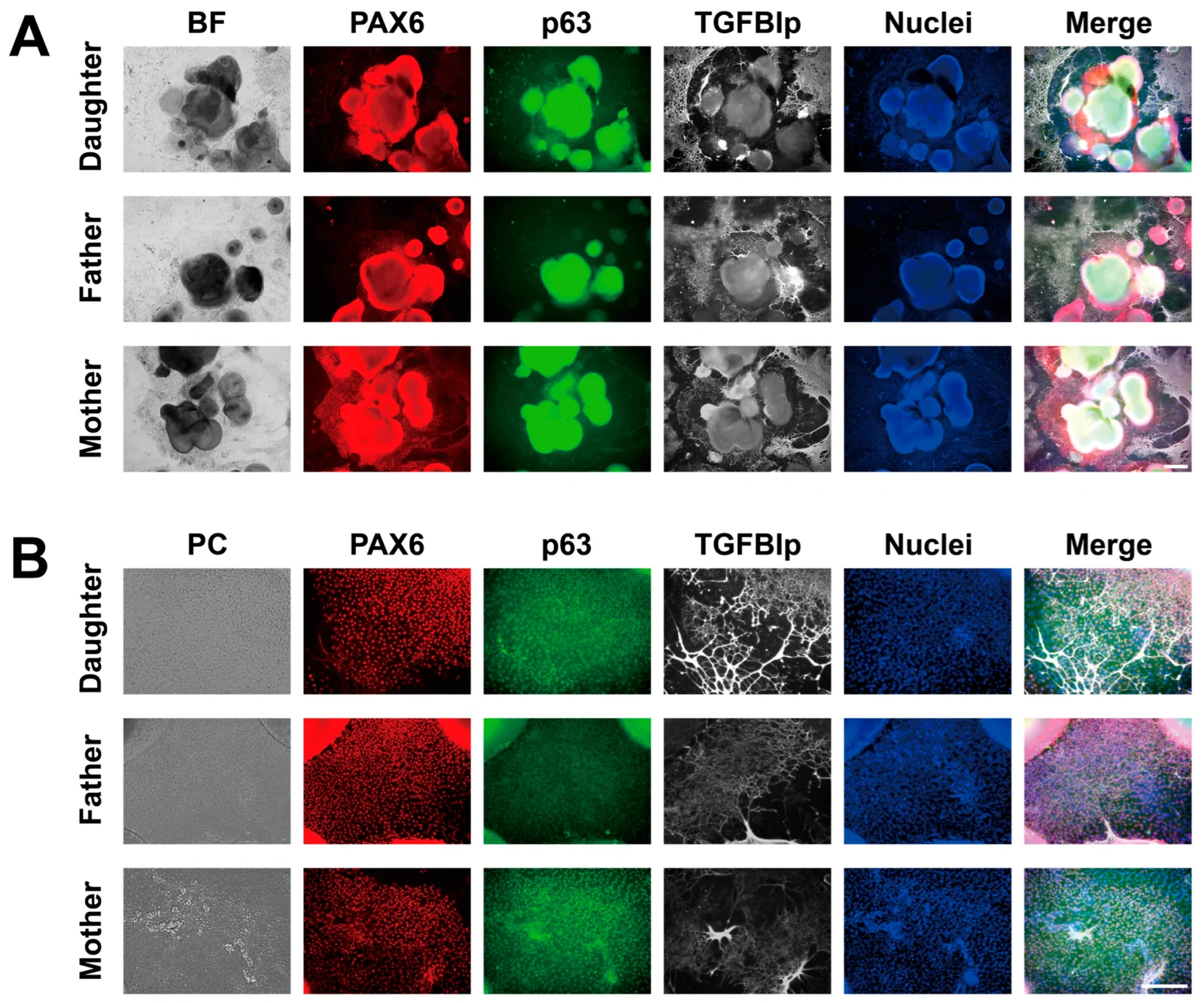
Immunofluorescence characterization of 4-week SEAM cultures derived from the RBCD family. Cultures of daughter-, father-, and mother-derived iPSC lines were stained for PAX6, p63, and TGFBIp, with Hoechst 33342 as a nuclear counterstain. Images are pseudocolored red (PAX6), green (p63), white (TGFBIp), and blue (nuclei): (**A**) Low-magnification images with corresponding bright-field (BF) images. Scale bar: 500 µm. (**B**) High-magnification images with corresponding phase-contrast (PC) images. Scale bar: 100 µm. Representative fields are shown.

Under SEAM induction conditions, early SEAM-like morphology was observed in the daughter-, father-, and mother-derived iPSC lines. After 4 weeks, PAX6- and p63-positive cells and extracellular/pericellular TGFBIp immunoreactivity were observed in all three lines, without a consistent qualitative difference in the examined staining patterns (Figure 5).

### 3.8. In Silico Structural and Surface Analysis of the TGFBI R124L Variant

**Figure 6:**
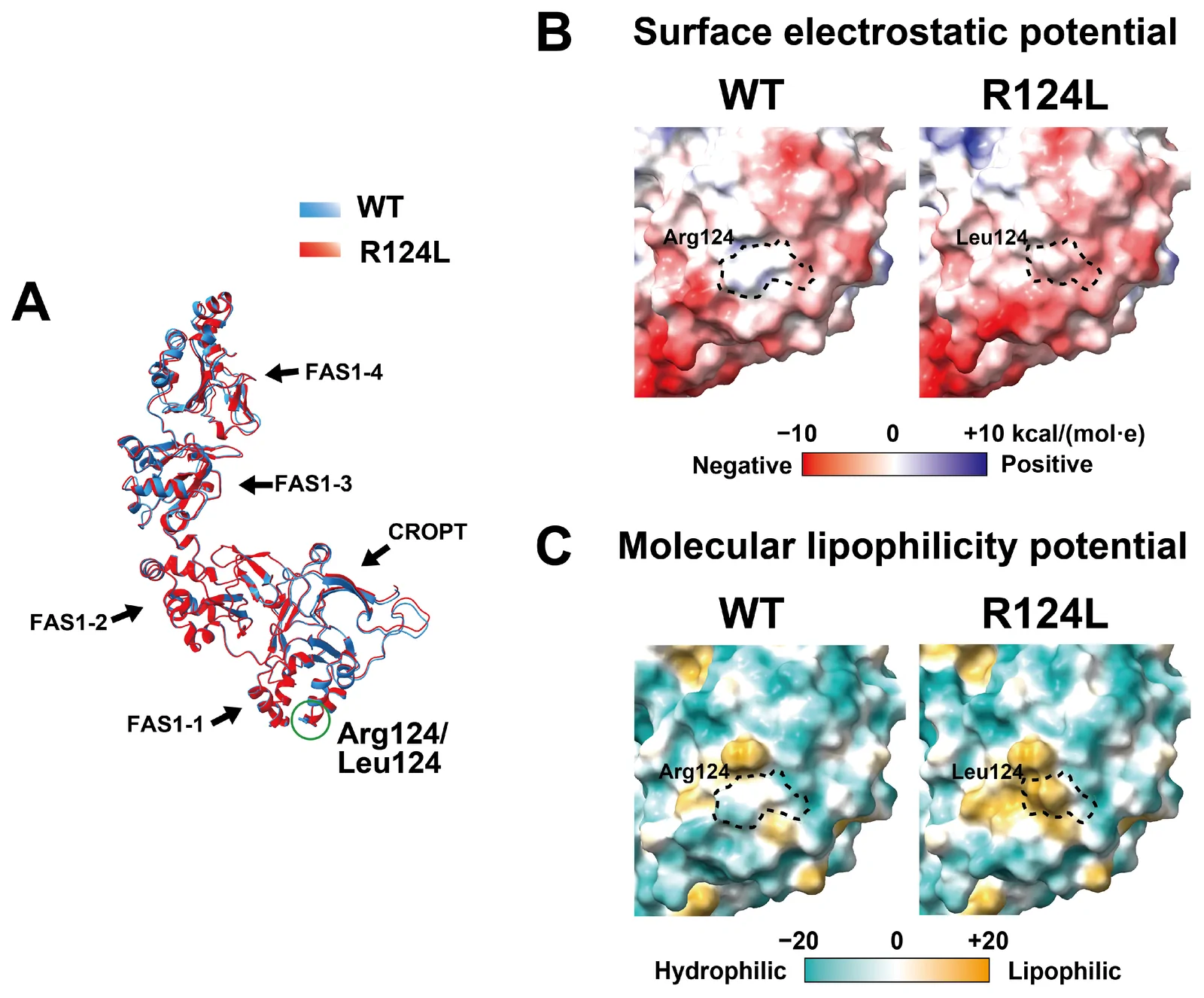
Structural comparison of WT and R124L TGFBIp and surface properties around residue 124: (**A**) Superposition of the cysteine-rich domain of periostin and TGFBIp (CROPT) and the four FAS1 domains from the top-ranked AlphaFold 3 models of wild-type (WT) TGFBIp and the R124L variant. WT and R124L are shown in blue and red, respectively. The CROPT (residues 45–99), FAS1-1 (residues 103–236), FAS1-2 (residues 240–371), FAS1-3 (residues 375–498), and FAS1-4 (residues 502–632) domains were superimposed using 576 corresponding Cα atoms. The Cα RMSD was 0.874 Å. Arg124 and Leu124 are indicated by the green circle. The N- and C-terminal regions were excluded because of their lower pLDDT values, whereas the short interdomain linkers were excluded to restrict the comparison to the defined domain regions. (**B**) Coulombic surface potential of energy-minimized WT and R124L models derived from chain A of PDB ID: 5NV6. Replacement of Arg124 with Leu reduced the positively charged surface area around residue 124 (dotted line). Electrostatic potentials were mapped using Amber 20 recommended charges and atom types over a range from −10 to +10 kcal/(mol·e); red, white, and blue indicate negative, neutral, and positive potential, respectively. (**C**) Molecular lipophilicity potential (MLP) of the same models. The surface around Leu124 was more lipophilic than the corresponding region around Arg124 in WT (dotted line). MLP was displayed over a range from −20 to +20; cyan, white, and yellow to orange indicate hydrophilic, neutral, and lipophilic regions, respectively.

In silico analyses were performed to compare the structures of WT and R124L TGFBIp and the surface properties around residue 124 (Figure 6).

The mean atom-level pLDDT values across the CROPT and four FAS1 domains were 92.5 for WT and 92.4 for R124L. In contrast, the mean pLDDT values for the N-terminal region were 62.8 for WT and 62.6 for R124L, whereas those for the C-terminal region were 48.1 and 50.8, respectively. The terminal regions were therefore excluded from the structural comparison. Superposition of the CROPT and four FAS1 domains from the top-ranked AlphaFold 3 models gave a Cα RMSD of 0.874 Å over 576 residue pairs (Figure 6A). The RMSDs obtained by separate superposition of each domain ranged from 0.105 to 0.209 Å. Residue 124 was located on the surface of FAS1-1.

In the energy-minimized 5NV6-based models, replacement of Arg124 with Leu reduced the positively charged surface area around residue 124 (Figure 6B). The surface around Leu124 was also more lipophilic than the corresponding region around Arg124 in WT (Figure 6C).

## 4. Discussion

### 4.1. Establishment of Family-Derived RBCD iPSC Lines

We established iPSC lines from an affected daughter, her affected father, and her unaffected mother. The two affected lines retained the *TGFBI* R124L variant. Basic characterization was satisfactory in all three lines: donor identity was confirmed, no karyotypic abnormalities were detected, and pluripotency marker expression and trilineage differentiation were demonstrated.

RBCD associated with R124L is clinically and pathologically distinct from other *TGFBI*-related corneal dystrophies [3,4,5,8,14,15]. The present family set includes two independently established affected lines carrying the same variant and an unaffected line from the same family. This study is the first to establish iPSC lines from an RBCD family carrying the TGFBI R124L variant and to demonstrate their early response to ocular surface induction, including the development of SEAM-like morphology. The mother-derived line is not isogenic with either affected line and is genetically related only to the daughter. It should therefore be regarded as a family control rather than a mutation-specific control.

### 4.2. Interpretation of Early SEAM-like Morphology and Marker Expression

All three lines developed early SEAM-like morphology under the induction conditions used in this study. Differentiation of human pluripotent stem cells toward ocular and corneal epithelial lineages has been reported to show cell-line-dependent variability [18,31]. Therefore, the observation that all three independently established family-derived iPSC lines exhibited early SEAM-like morphology under the same induction conditions supports their potential utility for subsequent ocular differentiation and disease-modeling studies. No obvious difference between the patient-derived and mother-derived lines was detected by phase-contrast microscopy. Qualitative examination of representative fields also revealed no consistent differences in PAX6, p63, or TGFBIp staining patterns among the three lines (Figure 5).

RBCD usually becomes clinically apparent during childhood, with recurrent corneal epithelial erosion followed by progressive subepithelial opacity. In the present proband, epithelial erosion began at 3 years of age, whereas the opacity progressed later.

However, the lack of an obvious difference after 4 weeks of SEAM induction does not exclude disease-related changes that may emerge during prolonged culture, potentially involving extracellular TGFBIp accumulation [8,9,10,14,15].

The present experiment evaluated early morphology and immunofluorescence staining for PAX6, p63, and TGFBIp. It did not establish mature corneal epithelial identity or stromal differentiation, and immunofluorescence alone did not demonstrate pathological TGFBIp deposition or quantify its secretion or turnover. Thus, the findings show that all three lines responded to SEAM induction, but they do not show that the patient-derived cells have a normal corneal phenotype. Longer culture and lineage-specific analyses will be needed to examine disease-related changes.

### 4.3. Relationship to TGFBIp Deposition and Aggregation

Deposition and aggregation of mutant TGFBIp are central features of *TGFBI*-related corneal dystrophies [2,3,14,15]. However, how individual variants produce different forms and distributions of extracellular deposits remains unresolved [14,15,16].

In the reported experimental structure of TGFBIp, residue 124 is located on the surface of the FAS1-1 domain [26]. Within the high-confidence regions analyzed, the predicted structures of the CROPT and four FAS1 domains were similar between the WT and R124L AlphaFold 3 models. However, the ability of AlphaFold-based methods to detect subtle structural changes caused by a single amino acid substitution has not been established [32,33]. In addition, pLDDT represents confidence in the predicted local structure [23] and does not indicate whether a difference, or lack of difference, between WT and mutant models reflects an actual mutation-induced structural effect. Therefore, the low RMSD observed in this study should not be interpreted as evidence that the R124L substitution has no structural effect. Rather, no major difference was detected between the high-confidence predicted domain structures. In the experimental structure-based surface analysis, replacement of the positively charged Arg124 with nonpolar Leu reduced the local positive surface charge and increased lipophilicity. Hydrophobic surface patches may contribute to protein association and aggregation in aqueous environments by reducing the exposure of apolar regions to water [34].

A previous structural study of the R124H and R555W variants identified an interface involving residue 124 that may contribute to dimerization and aggregation [35]. For R124L, it remains to be tested whether the altered surface changes interactions between TGFBIp molecules, binding to extracellular matrix components, or extracellular turnover. No experiment was performed in this study to determine whether the R124L-associated change in surface lipophilicity promotes TGFBIp aggregation. These questions can be examined directly by measuring TGFBIp secretion, degradation, and deposition in differentiated patient-derived cells.

### 4.4. Future Disease Modeling and Drug Evaluation

To model RBCD more directly, the next experiments should move beyond the present 4-week SEAM characterization. The present lines can be differentiated into corneal epithelial and stromal cells and maintained for longer periods. Corneal epithelial differentiation, epithelial–stromal co-culture, and three-dimensional corneal tissue systems have been reported and provide possible settings in which TGFBIp expression and extracellular deposition can be examined [18,19,36,37].

TGFBIp in the culture medium and extracellular matrix should be measured separately in epithelial and stromal cultures. Comparison with co-cultures would then help determine whether one cell type is mainly responsible for mutant TGFBIp production or whether epithelial–stromal interactions promote its accumulation. These experiments would address the origin of the deposits observed in RBCD.

Drug evaluation will become more informative after a reproducible TGFBIp deposition phenotype has been established. Approaches affecting TGFBIp expression, protein quality control, or degradation have been discussed for *TGFBI*-related corneal dystrophies [14,15,16,38,39]. The present lines could be used to examine such approaches once the relevant cellular phenotype has been defined. If candidate therapies are subsequently developed for topical administration, strategies to improve corneal drug delivery, such as nanocarrier-based approaches investigated in chemical corneal injury, may also be considered [40].

### 4.5. Limitations

This study has several limitations. First, the study was limited to a single family comprising two related affected individuals and one unaffected family member, with only one colony-derived iPSC line from each donor used for subsequent characterization. The three independent differentiation experiments therefore represent repeated observations of the same lines rather than independent donor- or genotype-level replication. The maternal line is not an isogenic control, and mutation-specific effects cannot be established from these comparisons. Second, the qPCR data were obtained from three independent differentiation experiments per line and are presented descriptively without formal statistical testing. ACTB was the sole reference gene, and its stability across differentiation states was not independently established (Supplementary Table S1). Amplification efficiencies were not determined using dilution-series standard curves. These limitations may affect the magnitudes of the calculated fold changes, particularly for small changes and low-abundance transcripts. Finally, the 4-week SEAM immunofluorescence analysis did not establish an RBCD-specific phenotype or directly measure TGFBIp secretion, turnover, or pathological deposition.

## 5. Conclusions

We established iPSC lines from an affected daughter and father carrying the *TGFBI* R124L variant and from an unaffected mother in the same family. The affected lines retained the variant, and all three lines showed pluripotency, trilineage differentiation, and early SEAM-like morphology. After 4 weeks of SEAM induction, PAX6- and p63-positive cells and extracellular/pericellular TGFBIp immunoreactivity were observed in all three lines.

The present study does not yet reproduce RBCD pathology. Its main contribution is the establishment of a family-based set of cells for direct studies of TGFBIp production and deposition in corneal epithelial and stromal lineages. The combination of two affected R124L-positive lines and an unaffected maternal line, together with their initial ocular differentiation characterization, provides a valuable foundation for future investigations of TGFBIp biology and RBCD disease modeling.

## Figures and Tables

**Table 1:** Characteristics of the study participants and established iPSC lines.

Participant	Age	Sex	Clinical Status	*TGFBI* Variant (NM_000358.3)	Mycoplasma
Daughter	12	Female	RBCD	c.371G>T [p.(Arg124Leu)] *, heterozygous	Negative
Father	45	Male	RBCD	c.371G>T [p.(Arg124Leu)] *, heterozygous	Negative
Mother	45	Female	Unaffected	Not detected	Negative

* The predicted protein consequence is based on NP_000349.1.

**Table 2:** Primer sets used for this study.

Assay	Category	Target Gene	Primer Direction	Primer Sequence (5′–3′)	Product Size (bp)
*TGFBI* genotyping	c.371G>T region	*TGFBI*	Forward	CCTCGTCCTCTCCACCTGTA	325
			Reverse	GGGAAGTAAGGCAGTTCCCC	
qPCR	Internal control	*ACTB*	Forward	ACAGAGCCTCGCCTTTGC	160
			Reverse	CACGATGGAGGGGAAGACG	
	Ectoderm	*PAX6*	Forward	CTGAGGAATCAGAGAAGACAGGC	131
			Reverse	ATGGAGCCAGATGTGAAGGAGG	
		*SOX1*	Forward	GCTGACACCAGACTTGGGTT	137
			Reverse	CCTCTCGCCTCGTTTTGACT	
	Endoderm	*SOX17*	Forward	TTCGTGTGCAAGCCTGAGAT	119
			Reverse	TAATATACCGCGGAGCTGGC	
		*FOXA2*	Forward	CCCCTACGCCAACATGAACT	125
			Reverse	GCGAGATGTACGAGTAGGGC	
	Mesoderm	*TBXT*	Forward	GCCAGTGGTGACTGGATTGA	228
			Reverse	CACCGTCAGTGAGGTTGGG	
		*TBX6*	Forward	TCATCTCCGTGACAGCCTACCA	137
			Reverse	CCGCAGTTTCCTCTTCACACGG	

**Table 3:** List of antibodies used in this study.

Antibody	Fluorochrome	Distributor	Clone	Dilution
**Flow cytometry and immunocytochemistry of iPSCs**				
NANOG	Alexa Fluor 647	BioLegend	23D2-3C6	1:10
SSEA-4	FITC	BioLegend	MC-813-70	1:25
TRA-1-60R	Alexa Fluor 647	BioLegend	TRA-1-60R	1:25
OCT4	Alexa Fluor 488	BioLegend	3A2A20	1:10
SOX2	Alexa Fluor 488	BioLegend	14A6A34	1:50
**Immunofluorescence staining of SEAM cultures: primary antibodies**				
p63	—	Nichirei Biosciences	4A4	Ready-to-use
PAX6	—	BioLegend	Poly19013	1:100
TGFBIp (βig-h3)	—	R&D Systems	Polyclonal	1:100
**Immunofluorescence staining of SEAM cultures: secondary antibodies**				
Donkey anti-mouse IgG	Alexa Fluor 488	Abcam	Polyclonal	1:200
Donkey anti-rabbit IgG	Alexa Fluor 647	Abcam	Polyclonal	1:200
Donkey anti-goat IgG	Alexa Fluor 568	Abcam	Polyclonal	1:200

**Table 4:** Short tandem repeat (STR) profiles at nine loci and amelogenin results of donor peripheral blood mononuclear cells and corresponding iPSC lines.

	Daughter				Father				Mother			
	PB		iPSC		PB		iPSC		PB		iPSC	
Marker	Allele 1	Allele 2	Allele 1	Allele 2	Allele 1	Allele 2	Allele 1	Allele 2	Allele 1	Allele 2	Allele 1	Allele 2
TH01	6	6	6	6	6	9	6	9	6	9	6	9
D21S11	31	31	31	31	29	31	29	31	31	31	31	31
D5S818	10	13	10	13	13	14	13	14	10	12	10	12
D13S317	10	11	10	11	10	11	10	11	10	10	10	10
D7S820	11	12	11	12	11	12	11	12	11	11	11	11
D16S539	9	12	9	12	11	12	11	12	9	12	9	12
CSF1PO	10	12	10	12	12	12	12	12	10	12	10	12
AMEL	X	X	X	X	X	Y	X	Y	X	X	X	X
vWA	14	17	14	17	14	14	14	14	14	17	14	17
TPOX	8	8	8	8	8	8	8	8	8	11	8	11
STR concordance	9/9 matched				9/9 matched				9/9 matched			

AMEL, amelogenin; PB, peripheral blood; iPSC, induced pluripotent stem cell.

## Data Availability

The data supporting the findings of this study are available from the corresponding authors upon reasonable request. Data containing clinical, genetic, or other potentially identifiable information are not publicly available because of ethical and privacy restrictions.

## Supplementary Materials

The following supporting information can be downloaded at https://www.mdpi.com/article/10.3390/cells15212025/s1, Figure S1: Representative flow cytometric gating strategies for SSEA-4 and TRA-1-60 analysis of donor-derived iPSCs; Table S1: ACTB Ct values across undifferentiated and trilineage-differentiated states; Table S2: Experiment-level ACTB and target-gene Ct values across undifferentiated and trilineage-differentiated states.

## Abbreviations

The following abbreviations are used in this manuscript:

7-AAD	7-aminoactinomycin D
CROPT	cysteine-rich domain of periostin and TGFBIp
DSCL	disposable soft contact lens
FBS	fetal bovine serum
FL	Flt3 ligand
G-CSF	granulocyte colony-stimulating factor
HCL	hard contact lens
IC3D	International Committee for Classification of Corneal Dystrophies
IL	interleukin
iPSC	induced pluripotent stem cell
ISCN	International System for Human Cytogenomic Nomenclature
MLP	molecular lipophilicity potential
MSC	mesenchymal stem cell
OCT	optical coherence tomography
PB	peripheral blood
PBMC	peripheral blood mononuclear cell
PBS	phosphate-buffered saline
PCR	polymerase chain reaction
PDB	Protein Data Bank
pLDDT	predicted local distance difference test
qPCR	quantitative polymerase chain reaction
RBCD	Reis–Bücklers corneal dystrophy
RMSD	root-mean-square deviation
SCF	stem cell factor
SEAM	self-formed ectodermal autonomous multizone
SFM	serum-free medium
STR	short tandem repeat
TGFBI	transforming growth factor beta-induced
TGFBIp	transforming growth factor beta-induced protein
TPO	thrombopoietin
WT	wild type
