# Supplementary material for: Establishment and Characterization of TGFBI R124L iPSCs Derived from a Reis–Bücklers Corneal Dystrophy Family
Source: Cells. 2026 Nov 6;15(21):2025. doi: 10.3390/cells15212025 (PMC13762281; doi:10.3390/cells15212025)
Supplement: Supplementary file 1 [file cells-15-02025-s001.zip › cells-4550937-supplementary.pdf]

Supplementary Materials

Supplementary Figure S1

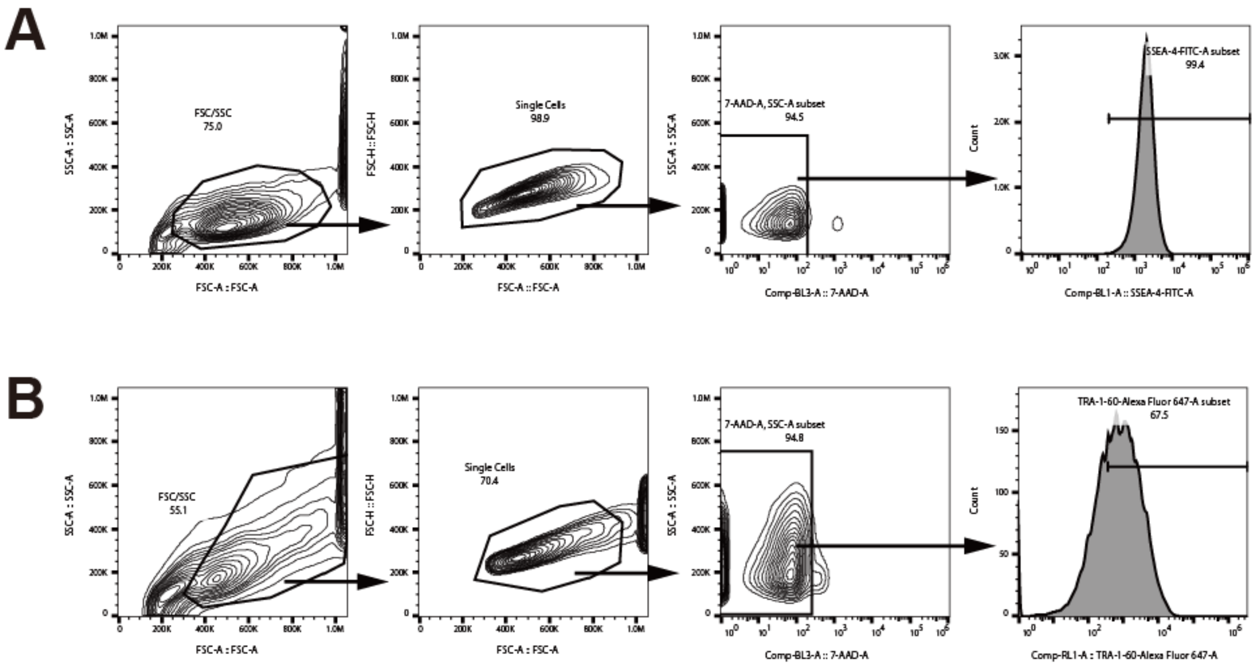

Supplementary Figure S1. Representative flow cytometric gating strategies for SSEA-4 and TRA-1-60 analysis of donor-derived iPSCs. (A) SSEA-4. (B) TRA-1-60. Cells were gated on forward scatter (FSC) and side scatter (SSC), followed by singlet discrimination and exclusion of 7-AAD-positive dead cells. Histograms show the proportion of marker-positive cells within the viable singlet population.

Supplementary Table S1. ACTB Ct values across undifferentiated and trilineage-differentiated states

Experiment	Donor-derived line	Undifferentiated iPSC	Ectoderm	Mesoderm	Endoderm	ACTB Ct range (max – min)
Exp. 1	Daughter	21.930	21.550	21.290	19.720	2.210
Exp. 1	Father	27.235	27.640	23.915	23.400	4.240
Exp. 1	Mother	22.255	23.500	21.625	21.440	2.060
Exp. 2	Daughter	19.510	19.255	19.620	18.885	0.735
Exp. 2	Father	19.025	18.190	19.625	21.895	3.705
Exp. 2	Mother	19.390	18.480	18.895	19.900	1.420
Exp. 3	Daughter	20.405	19.735	20.405	19.125	1.280
Exp. 3	Father	20.255	21.050	19.830	19.865	1.220
Exp. 3	Mother	19.870	22.475	20.235	19.120	3.355

Values represent the mean ACTB Ct of two technical qPCR replicates for each cell state. The ACTB Ct range was calculated as the difference between the maximum and minimum ACTB Ct values across the four cell states for each donor-derived line within each experiment.

Supplementary Table S2. Experiment-level ACTB and target-gene Ct values across undifferentiated and trilineage-differentiated states

Experiment	Donor-derived line	Gene	Undifferentiated iPSC	Ectoderm	Mesoderm	Endoderm
Exp. 1	Daughter	ACTB	21.930	21.550	21.290	19.720
Exp. 1	Father	ACTB	27.235	27.640	23.915	23.400
Exp. 1	Mother	ACTB	22.255	23.500	21.625	21.440
Exp. 1	Daughter	PAX6	29.955	18.560	29.620	23.150
Exp. 1	Father	PAX6	32.580	21.900	31.110	26.125
Exp. 1	Mother	PAX6	31.010	20.235	29.970	26.410
Exp. 1	Daughter	SOX1	29.230	22.725	27.980	28.350
Exp. 1	Father	SOX1	29.805	27.040	29.445	28.970
Exp. 1	Mother	SOX1	30.325	24.430	29.475	31.090
Exp. 1	Daughter	SOX17	30.320	31.805	29.005	19.100
Exp. 1	Father	SOX17	30.775	30.095	30.285	21.545
Exp. 1	Mother	SOX17	31.410	33.790	30.110	23.250
Exp. 1	Daughter	FOXA2	32.430	38.070	30.405	24.485
Exp. 1	Father	FOXA2	32.525	31.930	31.425	26.165
Exp. 1	Mother	FOXA2	33.505	36.225	31.960	28.405
Exp. 1	Daughter	TBXT	31.960	32.895	19.825	25.770
Exp. 1	Father	TBXT	34.580	33.080	21.240	27.145
Exp. 1	Mother	TBXT	31.905	36.415	20.495	28.165
Exp. 1	Daughter	TBX6	29.630	28.460	22.450	28.775
Exp. 1	Father	TBX6	32.805	31.980	24.300	30.425
Exp. 1	Mother	TBX6	30.335	30.145	23.915	30.680

Experiment	Donor-derived line	Gene	Undifferentiated iPSC	Ectoderm	Mesoderm	Endoderm
Exp. 2	Daughter	ACTB	19.510	19.255	19.620	18.885
Exp. 2	Father	ACTB	19.025	18.190	19.625	21.895
Exp. 2	Mother	ACTB	19.390	18.480	18.895	19.900
Exp. 2	Daughter	PAX6	29.060	17.585	29.545	22.930
Exp. 2	Father	PAX6	28.905	18.110	29.745	24.235
Exp. 2	Mother	PAX6	28.485	18.320	28.010	23.875
Exp. 2	Daughter	SOX1	28.785	20.930	27.815	28.060
Exp. 2	Father	SOX1	28.185	22.820	29.435	28.750
Exp. 2	Mother	SOX1	28.265	22.540	27.660	28.250
Exp. 2	Daughter	SOX17	30.200	33.115	29.080	18.775
Exp. 2	Father	SOX17	30.220	29.840	29.700	18.975
Exp. 2	Mother	SOX17	30.265	31.105	28.755	20.525
Exp. 2	Daughter	FOXA2	32.275	34.320	29.565	22.530
Exp. 2	Father	FOXA2	31.535	31.155	29.305	22.960
Exp. 2	Mother	FOXA2	31.875	33.275	30.205	24.240
Exp. 2	Daughter	TBXT	29.820	34.340	19.295	25.400
Exp. 2	Father	TBXT	29.340	31.395	18.705	25.395
Exp. 2	Mother	TBXT	28.670	32.660	18.710	25.220
Exp. 2	Daughter	TBX6	27.090	25.840	20.105	26.645
Exp. 2	Father	TBX6	26.310	26.510	20.345	26.290
Exp. 2	Mother	TBX6	26.255	26.335	20.555	26.055

Experiment	Donor-derived line	Gene	Undifferentiated iPSC	Ectoderm	Mesoderm	Endoderm
Exp. 3	Daughter	ACTB	20.405	19.735	20.405	19.125
Exp. 3	Father	ACTB	20.255	21.050	19.830	19.865
Exp. 3	Mother	ACTB	19.870	22.475	20.235	19.120
Exp. 3	Daughter	PAX6	28.940	17.840	28.985	23.425
Exp. 3	Father	PAX6	29.680	18.635	29.870	24.575
Exp. 3	Mother	PAX6	28.215	19.090	28.400	24.290
Exp. 3	Daughter	SOX1	29.065	21.365	28.175	28.445
Exp. 3	Father	SOX1	28.085	23.420	29.505	28.240
Exp. 3	Mother	SOX1	28.685	22.770	25.505	28.385
Exp. 3	Daughter	SOX17	29.845	31.545	28.870	19.130
Exp. 3	Father	SOX17	30.055	29.420	29.575	19.515
Exp. 3	Mother	SOX17	30.065	31.420	29.165	20.745
Exp. 3	Daughter	FOXA2	32.630	33.340	30.585	23.325
Exp. 3	Father	FOXA2	32.520	32.835	30.435	23.670
Exp. 3	Mother	FOXA2	32.310	33.260	31.090	25.110
Exp. 3	Daughter	TBXT	31.330	35.110	20.410	26.385
Exp. 3	Father	TBXT	30.060	30.540	20.295	26.360
Exp. 3	Mother	TBXT	29.400	32.445	19.875	25.980
Exp. 3	Daughter	TBX6	27.255	26.685	20.935	27.340
Exp. 3	Father	TBX6	27.310	27.140	21.190	27.425
Exp. 3	Mother	TBX6	26.765	27.125	21.680	27.045

Values are means of two technical qPCR Ct replicates (three independent differentiation experiments per line); no values were excluded.

Values are unnormalized and were not corrected for RNA input or reverse-transcription efficiency.
